# The Antihistamine Deptropine Induces Hepatoma Cell Death through Blocking Autophagosome-Lysosome Fusion

**DOI:** 10.3390/cancers12061610

**Published:** 2020-06-18

**Authors:** Yu-Chih Liang, Chi-Ching Chang, Ming-Thau Sheu, Shyr-Yi Lin, Chia-Chen Chung, Chang-Ting Teng, Fat-Moon Suk

**Affiliations:** 1 School of Medical Laboratory Science and Biotechnology, College of Medical Science and Technology, Taipei Medical University, Taipei 11031, Taiwan; ycliang@tmu.edu.tw (Y.-C.L.); shettoangel@gmail.com (C.-C.C.); m609106009@tmu.edu.tw (C.-T.T.); 2 Ph.D. Program in Medical Biotechnology, College of Medical Science and Technology, Taipei Medical University, Taipei 11031, Taiwan; 3 Traditional Herbal Medicine Research Center, Taipei Medical University Hospital, Taipei 11031, Taiwan; 4 Department of Internal Medicine, School of Medicine, College of Medicine, Taipei Medical University, Taipei 11031, Taiwan; ccchang@tmu.edu.tw (C.-C.C.); sylin@tmu.edu.tw (S.-Y.L.); 5 Division of Rheumatology, Immunology and Allergy, Taipei Medical University Hospital, Taipei 11031, Taiwan; 6 School of Pharmacy, College of Pharmacy, Taipei Medical University, Taipei 11031, Taiwan; mingsheu@tmu.edu.tw; 7 Division of Gastroenterology, Department of Internal Medicine, Wan Fang Hospital, Taipei Medical University, Taipei 11696, Taiwan

**Keywords:** antihistamine, deptropine, hepatoma, autophagy, LC3B, SQSTM1/p62

## Abstract

Some antihistamines have exhibited significant antitumor activity alone or in combination with other therapies in in vitro and clinical studies. However, the underlying mechanisms of how antihistamines inhibit hepatocellular carcinoma proliferation are still unknown. We first screened the antiproliferation activity of 12 benzocycloheptene structural-analogue drugs, and results showed that deptropine was the most potent inhibitor of both Hep3B and HepG2 human hepatoma cells. Deptropine significantly increased light chain 3B-II (LC3B-II) expression but did not induce sequestosome 1 (SQSTM1/p62) degradation in either cell line. Interestingly, other autophagy-related proteins, such as autophagy-related 7 (ATG7), vacuolar protein sorting 34 (VPS34), phosphorylated adenosine 5′-monophosphate-activated protein kinase (AMPK), and phosphorylated protein kinase B (PKB, also known as Akt), exhibited no significant change in either deptropine-treated cell line. Deptropine also inhibited the processing of cathepsin L from its precursor form to its mature form. Immunofluorescence microscopy showed an increase of autophagosomes in deptropine-treated cells, but deptropine blocked the fusion between autophagosomes and lysosomes. In a xenograft nude mice model, 2.5 mg/kg deptropine showed a great inhibitory effect on Hep3B tumor growth. These results suggest that deptropine can induce in vitro and in vivo hepatoma cell death, and the underlying mechanisms might be mediated through inhibiting autophagy by blocking autophagosome-lysosome fusion.

## 1. Introduction

Hepatocellular carcinoma (HCC), also known as liver cancer, is one of the most common malignant tumors of the liver. It has higher incidences in Asian countries, such as Taiwan, China, and Japan, and relatively low incidences in Western countries. HCC is the second leading cause of cancer-related deaths in Taiwan and the fourth leading cause of cancer-related deaths worldwide [1,2]. The main causes of HCC are related to hepatitis B, hepatitis C, alcoholic liver disease, non-alcoholic fatty liver disease, and cirrhosis [3,4]. HCC is frequently asymptomatic in its early stages, and about 85% of patients diagnosed with HCC are in intermediate or advanced stages. Only surgical resection and liver transplantation are curative treatments for these patients. At present, there is no ideal chemotherapeutic drug to treat patients with HCC.

Recently, the combination of cyproheptadine, an antihistamine drug, and thalidomide was reported to result in the disappearance of liver tumors and lung metastasis [5]. Another clinical study also demonstrated that sorafenib, a first-line drug for HCC treatment, combined with cyproheptadine increased the mean survival time of patients from 4.8 to 11 months, and the progression-free survival from 1.7 to 7.5 months [6]. Cyproheptadine alone significantly improved survival rates in patients with HCC compared to traditional therapies [7]. In a cell model experiment, cyproheptadine blocked cell cycle progression through activation of p38 mitogen-activated protein kinase (MAPK) in HCC cells, and resulted in inhibition of cell proliferation and apoptosis [8]. Several other antihistamine drugs were also demonstrated to have antitumor activity through various kinds of molecular mechanisms in cells or animal model experiments. Astemizole targets the ether à-go-go-1 (Eag1) potassium channel that is overexpressed in human HCC and ultimately inhibited cell proliferation and induced apoptosis [9]. Two other antihistamines, clemastine and desloratadine, induced T-cell lymphoma cell apoptosis that are involved in downregulating signal transducer and activator of transcription 3 (STAT3) and cellular myelocytomatosis (c-Myc) activities [10]. Moreover, ketotifen decreased the cell migration and invasion of breast cancer and fibrosarcoma cells, and the underlying mechanisms were associated with inhibition of cell division cycle 42 (Cdc42), Rho, Rac, and matrix metalloproteinase (MMP)-9 expressions [11]. Loratadin, a long-acting, non-sedating antihistamine drug enhanced the radiation sensitivity and then disrupted the cell cycle progression of human colon carcinoma cells [12]. Nortriptyline, a tricyclic antidepressant with some antihistamine H1-blocking activity, exhibited antitumor growth effects in bladder cancer cells. It induced both intrinsic and extrinsic apoptotic pathways, including upregulation of Fas, Fas ligand (FasL), Fas-associated protein with death domain (FADD), Bax, Bak, and cleaved forms of caspase-3, caspase-8, caspase-9, and poly (adenosine diphosphate-ribose) polymerase (PARP) [13]. These research results indicate that antihistamines play important roles in cancer treatment and have potential to be developed as new anticancer drugs.

Autophagy is a highly conserved intracellular degradation pathway that continually digests cytoplasmic proteins and organelles at the basal level of all cells [14,15,16]. In liver cells, autophagy maintains energy and nutrient balance, and a defect in autophagy genes can induce cell death under nutrient deprivation [17,18]. However, overactivation of autophagy leads to excessive catabolism and might induce the apoptotic machinery. It was proposed that both inhibition and enhancement of autophagy are therapeutic strategies for advanced cancers [19,20]. Five distinct stages of the autophagy process include initiation, elongation, maturation, fusion, and degradation [21]. Previous studies demonstrated that autophagic flux can be detected by measuring levels of light chain 3B-II (LC3-II) and sequestosome 1 (SQSTM1/p62). During the initiation to maturation stages of autophagy, LC3 is conjugated to phosphatidylethanolamine (PE) to form LC3-II which can be used as a marker of autophagosomes. When autophagy proceeds to the fusion and degradation stages, the cargos, as well as LC3 and the LC3-binding protein, SQSTM1/p62, are selectively degraded in autolysosomes [22]. Each stage has the potential to be a target for clinical cancer treatment, and many inhibitors for each stage are currently under preclinical development. Chloroquine and hydroxychloroquine, inhibitors of the fusion of autophagosomes and lysosomes, are under investigation in clinical trials [23].

According to recent publications, there is strong evidence that antihistamines can fight cancer, including in cell experiments, animal experiments, and even human clinical trials. However, the underlying molecular mechanisms of antihistamines in inhibiting HCC cell proliferation remain unclear. This study may provide a novel strategy for treating HCC in the future.

## 2. Results

### 2.1. Deptropine Was the Most Effective Drug against Hepatoma Cell Proliferation

**Figure 1:**
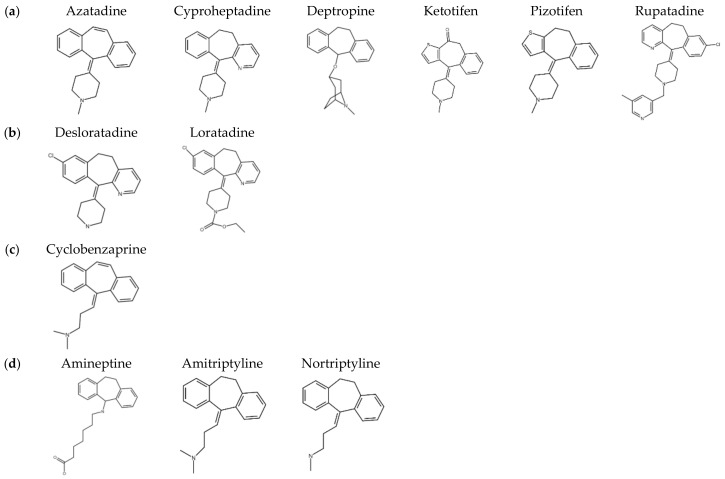
Structures of 12 benzocycloheptene analogous drugs. These drugs can be divided into four groups: (**a**) first-generation antihistamines, (**b**) second-generation antihistamines, (**c**) muscle relaxants, and (**d**) antidepressants and anticholinergics.

**Figure 2:**
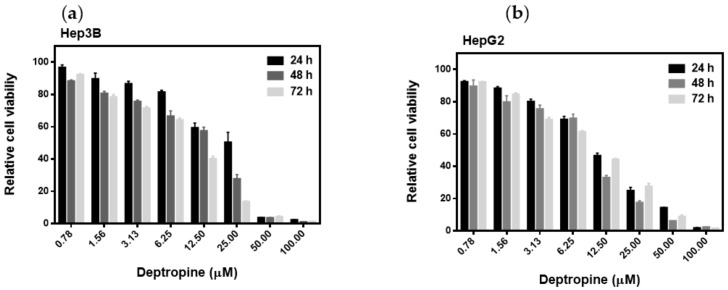
Dose- and time-dependent effects of deptropine on the cell viability of human hepatoma cells. (**a**) Hep3B and (**b**) HepG2 cells were treated with different concentrations of deptropine for 24, 48, or 72 h, and cell viability was determined by an 3-(4,5-Dimethylthiazol-2-yl)-2,5-Diphenyltetrazolium Bromide (MTT) assay. Data are presented as the mean ± standard error of three independent experiments.

Previous studies demonstrated that the antihistamine, cyproheptadine, had antitumor activity both in in vitro and in vivo [5,6,7]. The backbone structure of cyproheptadine is a benzocycloheptene, and structurally analogous drugs are widely used in the clinic, such as antihistamines, anticholinergics, antidepressants, and antiserotonergics. The cytotoxicities toward both Hep3B and HepG2 human hepatoma cells of a series of benzocycloheptenes structural analogue drugs were tested, including first-generation antihistamines, such as azatadine, cyproheptadine, deptropine, ketotifen, pizotifen, and rupatadine; second-generation antihistamines, such as desloratadine and loratadine; the muscle relaxant, cyclobenzaprine; antidepressants, and anticholinergics, such as amineptine, amitriptyline, and nortriptyline (Figure 1). As shown in Table 1, deptropine was the most potent inhibitor of proliferation by both cells compared to the other benzocycloheptene structurally analogous drugs. The 50% inhibitory concentration (IC_50_) values of deptropine were as low as 9.98 ± 0.12 and 9.75 ± 0.11 μM in Hep3B and HepG2 cells, respectively. Rupatadine and nortriptyline also exhibited strong inhibition of proliferation by both cells with IC_50_ values of approximately 13 μM. Azatadine and amineptine were the least effective drugs with IC_50_ values of >50 μM, and the other drugs also exhibited cytotoxicity effects with IC_50_ values between 20 and 40 μM in both cell lines. In addition, deptropine inhibited cell proliferation in dose- and time-dependent manners (Figure 2). These results suggested that deptropine and several other benzocycloheptene structural analogue drugs have cytotoxic activity against human hepatoma cells. This section may be divided by subheadings. It should provide a concise and precise description of the experimental results and their interpretation, as well as the experimental conclusions that can be drawn.

### 2.2. Deptropine Induced Autophagosome Formation but Did Not Cause Degradation of the SQSTM1/p62 Autophagic Substrate

**Figure 3:**
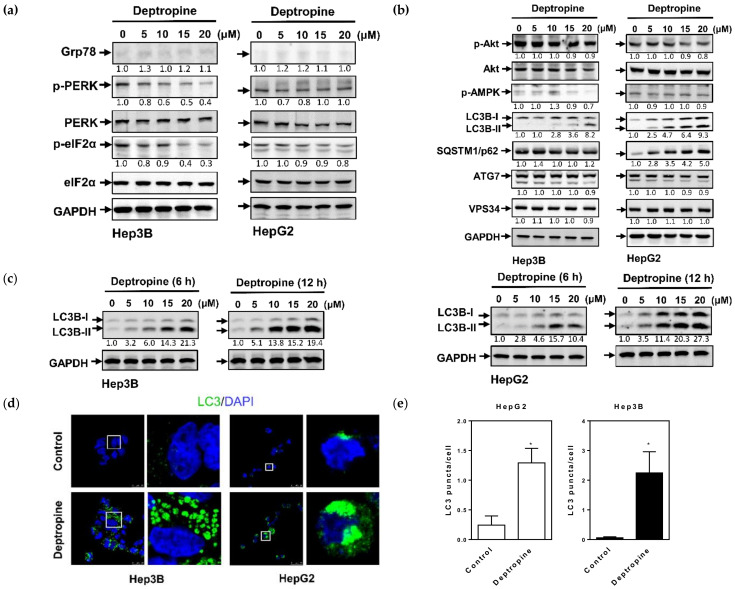
Effects of deptropine on marker protein expressions of endoplasmic reticular stress and autophagy, as well as the formation of light chain 3 (LC3) puncta, in human hepatoma cells. (**a**,**b**) Hep3B and HepG2 cells were treated with different concentrations of deptropine for 48 h, and the marker protein expressions of (**a**) endoplasmic reticular stress and (**b**) autophagy were determined by Western blotting. The relative intensity of each band (indicated below the bands) was normalized to the total protein (including protein kinase RNA-like endoplasmic reticulum kinase (PERK), eukaryotic translation initiation factor 2 subunit alpha (eIF2α), and protein kinase B (PKB, also known as Akt) or glyceraldehyde 3-phosphate dehydrogenase (GAPDH) loading control. Grp78, glucose-regulated protein 78; AMPK, adenosine 5′-monophosphate-activated protein kinase; SQSTM1, sequestosome-1; ATG7, autophagy related 7; VPS34, vacuolar protein sorting 34. (**c**) Hep3B and HepG2 cells were treated with different concentrations of deptropine for 6 or 12 h, and light chain 3B (LC3B) expression was determined by Western blotting. The relative intensity of LC3B-II was normalized to the GAPDH loading control and indicated below the bands. (**d**,**e**) HepG2 and Hep3B cells were treated with 20 μM of deptropine for 24 h, and LC3 puncta were detected by immunofluorescence staining with an LC3-specific antibody (green) and nucleic acid staining with 4′,6-diamidino-2-phenylindole (DAPI) (blue). (**d**) Representative immunofluorescence images are shown, and (**e**) quantification of LC3 puncta per cell is presented as the mean ± standard error. * *p* < 0.01.

To investigate the molecular mechanisms of deptropine in inhibiting hepatoma cell proliferation, we first examined whether deptropine could induce endoplasmic reticular (ER) stress in human hepatoma cells. The expression of glucose-regulated protein 78 (Grp78), which plays a role as a gatekeeper in activating ER stress, and phosphorylation of protein kinase RNA-like endoplasmic reticulum kinase (PERK) and eukaryotic translation initiation factor 2 subunit alpha (eIF2α), which are ER-specific signals of ER stress, were measured by Western blot analyses. As shown in Figure 3a, deptropine did not induce Grp78 expression but decreased the phosphorylation of PERK and eIF2α in both hepatoma cell lines (Figure S1). Results suggested that deptropine was unable to induce ER stress in hepatoma cells. Next, we examined whether deptropine could induce cell death through regulating autophagy. As shown in Figure 3b, LC3B-II expression had increased in both cells after treatment with deptropine for 48 h (Figure S2). Increases of LC3B-II were found as early as 6 and 12 h of treatment with deptropine (Figure 3c and Figure S3). However, the autophagic substrate, SQSTM1/p62, was not degraded in either deptropine-treated cell line and even increased in deptropine-treated HepG2 cells, indicating that autophagy progression might be incomplete. Other autophagy regulators, such as Akt, phosphorylated adenosine 5′-monophosphate-activated protein kinase (AMPK), and vacuolar protein sorting 34 (VPS34), as well as the autophagy-associated regulatory factor, ATG7, elicited no change in either deptropine-treated cell line. In addition, we also examined whether deptropine could induce autophagosome formation by immunocytochemical staining with the LC3B antibody. As shown in Figure 3d,e, deptropine significantly increased the number of LC3B puncta in both cell lines. These results suggest that deptropine might induce autophagosome formation but arrest autophagy progression.

### 2.3. Deptropine Blocked Basal Autophagy through Blocking Autophagosome and Lysosome Fusion

**Figure 4:**
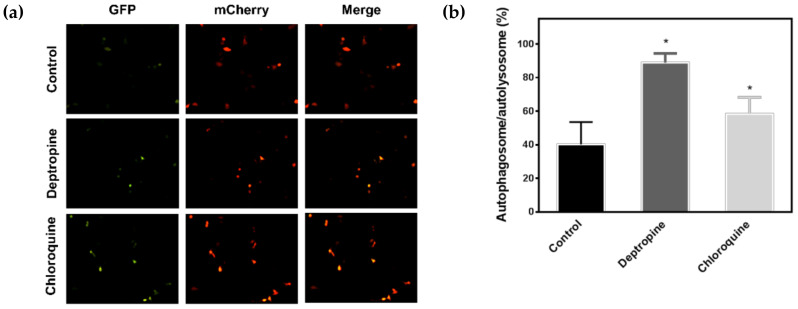
Effects of deptropine on inhibition of autophagosome-lysosome fusion in human hepatoma cells. Hep3B cells were transfected with the FUW mCherry-green fluorescent protein (GFP)-LC3 plasmid and then treated with 20 μM of deptropine for 24 h. Autophagosomes/autolysosomes were visualized with fluorescence microscopy. Chloroquine (25 μΜ) was used as a positive control. (**a**) Representative fluorescence images are shown, and (**b**) autophagic flux was estimated as the ratio between yellow-positive cells and red-positive cells, which are presented as the mean ± standard error. * *p* < 0.0001.

**Figure 5:**
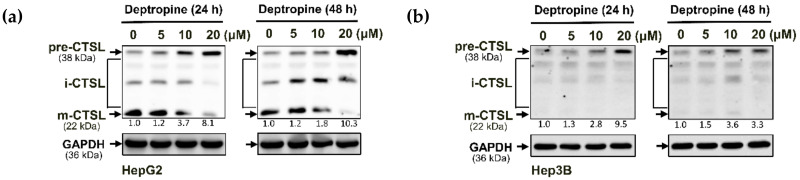
Effects of deptropine on the lysosomal activity of human hepatoma cells. (**a**) Hep3B and (**b**) HepG2 cells were treated with different concentrations of deptropine for 24 and 48 h. Total cell lysates were collected, and the precursor form (pre-), intermediate (i-), and mature (m-) form of the cathepsin L (CTSL) protein were detected by Western blotting. The relative intensity of pre-CTSL was normalized to the GAPDH loading control and indicated below the bands.

To understand why the SQSTM1/p62 protein accumulated in HepG2 cells and was not degraded in Hep3B cells, we investigated whether deptropine could block autophagic flux in hepatoma cells by transfecting cells with the mCherry-green fluorescent protein (GFP)-LC3 tandem reporter plasmid. In the neutral pH condition of autophagosomes, both the mCherry protein and GFP retained their red and green fluorescence, respectively, resulting in yellow fluorescence when merged. However, GFP loses its green fluorescence in the acidic condition of autophagolysosomes, resulting in red fluorescence of the LC3 protein. Therefore, the mCherry-GFP-LC3 protein can be used to measure changes in autophagic flux based on the ratio of yellow to red fluorescence. As shown in Figure 4, deptropine and chloroquine (the positive control) significantly increased the level of yellow fluorescence, suggesting that deptropine blocked autophagic flux. It is known that the mature form of cathepsin L (CTSL) can be used as a marker for lysosomal activity [24], and normal functioning of lysosomes is important for autophagosome-lysosome fusion [25]. We next examined whether deptropine could affect processing of the CTSL protein from the precursor form to its mature form. As shown in Figure 5, deptropine markedly increased levels of the precursor form of CTSL in both cell lines (Figure S4). These results suggest that deptropine initiated autophagy but failed to complete the maturation of autophagy by disrupting autophagosome-lysosome fusion.

### 2.4. Deptropine Induced Limited Caspase Activation

**Figure 6:**
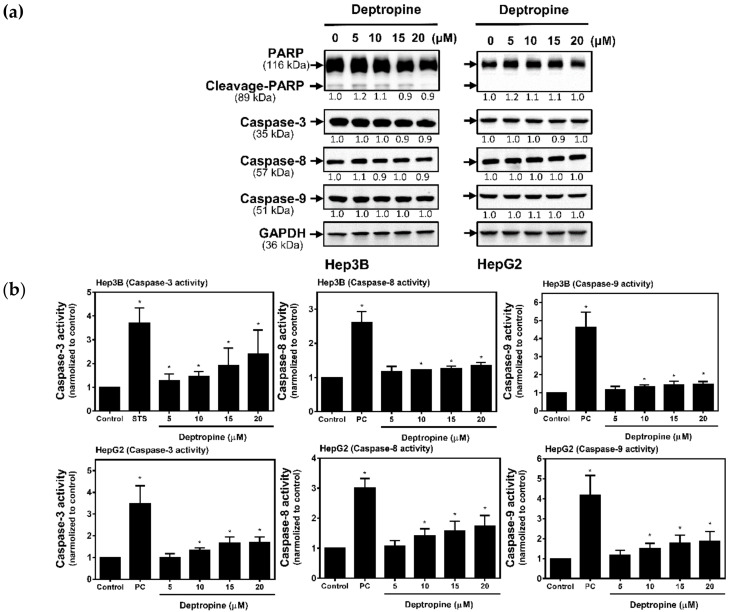
Effects of deptropine on caspase expressions and activities in human hepatoma cells. Hep3B and HepG2 cells were treated with different concentrations of deptropine for 48 h, and (**a**) poly(ADP ribose) polymerase (PARP), caspase-3, -8, and -9 protein expressions were determined by Western blotting. The relative intensity of each band (indicated below the bands) was normalized to the GAPDH loading control. (**b**) The cell extracts were subjected to caspase-3, -8, and -9 activity assays as described in “Materials and Methods”. Values were obtained in two independent experiments performed in triplicate, and results are presented as the mean ± standard error. * *p* < 0.05 vs. the control. STS, staurosporine (1 μM) as the positive control.

It has been known that the cross-talk between autophagy and apoptosis might occur in drug-induced cell death. To understand whether deptropine could also activate apoptosis, we next examined the cleavage of PARP and activation of caspases in deptropine-treated cells. After treatment with deptropine for 48 h, there was no decrease in the full lengths of PARP, caspase-3, -8, or -9, and no cleaved form of PARP according to a Western blot analysis (Figure 6a and Figure S5). However, the sensitivity of using Western blotting for detecting the cleaved forms of caspases is very low. To further verify whether deptropine can activate caspase cascades, we used caspase-3, -8, and -9 colorimetric assay kits to measure their activities. As shown in Figure 6b, deptropine significantly but slightly activated caspase-3, -8, and -9 activities compared to staurosporine (STS as the positive control). These results suggest that deptropine caused hepatoma cell death partly through activation of apoptosis.

### 2.5. Deptropine Inhibited Hepatoma Tumor Growth in Nude Mice

**Figure 7:**
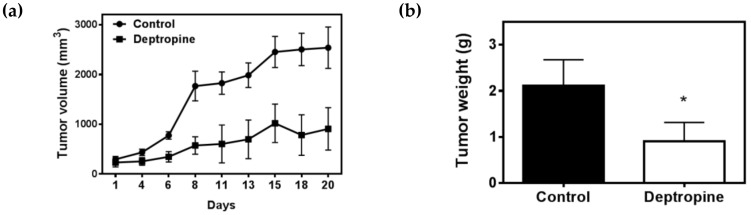
Effects of deptropine on hepatoma tumor xenografts in nude mice. Hep3B cells were subcutaneously injected between the scapulas of athymic nude mice, and the mice received an intraperitoneal (i.p.) injection of 2.5 mg/kg deptropine three times a week for 3 weeks. (**a**) The tumor volume was measured every 2 or 3 days, and (**b**) the tumor weight was measured at the end of the experiment. Values were obtained from seven or eight samples, and results are presented as the mean ± standard error. * *p* < 0.05 vs. the control.

To further examine the therapeutic effects of deptropine against hepatoma cells in vivo, we used a nude mice xenograft model. Athymic mice bearing Hep3B tumor xenografts were treated with 2.5 mg/kg deptropine three times a week for 3 weeks. Tumor volumes were measured every 2 or 3 days, and the body weight and tumor weight were measured at the end of the experiment. As shown in Figure 7, 2.5 mg/kg of deptropine significantly inhibited the tumor volume and tumor weight by about 64.2% and 57.3%, respectively, compared to control tumors, and body weights remained unchanged between control and drug-treated mice. These results suggest that deptropine might have significant applications for hepatoma therapeutic purposes.

## 3. Discussion

Among 12 benzocycloheptene structurally analogous drugs, deptropine was the most effective in increasing HCC cell death. We found that deptropine increased LC3B-II expression and the number of autophagosomes, but failed to degrade SQSTM1/p62 by blocking the fusion of autophagosomes and lysosomes, and finally caused the death of hepatoma cells. Deptropine also significantly inhibited HCC tumor growth in a xenograft nude mice model. However, cell death did not seem to be the same as apoptosis, because there was very limited activation of caspases in deptropine-treated cells. These results suggest that deptropine can induce cell death through interfering with the process of autophagy in HCC.

**Figure 8:**
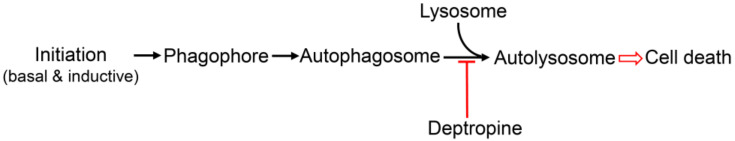
The possible mechanisms of deptropine-induced cell death in human hepatoma cells.

As mentioned in the "Introduction", basal autophagy occurs in all cells, and inhibition of basal autophagy or overactivation of autophagy can result in cell death. In this study, deptropine might not interfere with basal autophagy or overactivate autophagy but ultimately caused cell death due to blocking autophagosome and lysosome fusion (Figure 8). However, further investigations are needed to understand if deptropine either interferes with basal autophagy or overactivates autophagy in hepatoma cells.

Both human Hep3B and HepG2 hepatoma cells were used in this study; however, some results were inconsistent in both cells, such as the SQSTM1/p62 expression in deptropine-treated cells (Figure 3b). The inconsistent results might result from the different originations and genetic backgrounds of HepG2 and Hep3B cells, especially because Hep3B is hepatitis B virus positive but HepG2 is negative [26]. In addition, both cells have differential gene expression and differential drug responses of signaling pathways [26] that finally produced different responses for deptropine treatment. Deptropine has not been found to have significant hepatotoxicity in the literature, and the morphology and size of liver had no notable change in the deptropine-treated mice of this study. As we know, the cancer cells need to synthesize more amount of proteins for the demand of rapid growth and division. In the same way, the more unfolded and misfolded proteins possibly accumulate in cancer cells than in normal cells. The accumulation of unfolded and misfolded proteins triggers unfolded protein response (UPR) to relieve endoplasmic reticulum (ER) stress and then restore ER homeostasis. The autophagy induced by ER stress is considered to play an important role in the degradation of unfolded and misfolded proteins [27]. However, the process of autophagy was eventually blocked by deptropine, so the more cytotoxicity found in liver cancer cells was higher than that of normal liver cells in this study.

Antihistamines act as inverse agonists that more favorably bind to the inactive state of the histamine receptor (HR), which belongs to the G protein-coupled receptor (GPCR) family, and consequently downregulates the activity of the GPCR [28]. HRs are expressed in several human normal and tumor cells and tissues; for example, hepatocytes express the H1, H2, and H3 HRs [29]. All four types of HRs may be expressed in human and mouse hepatoma cells, and the H4 HR is increased in human HCC primary tumors compared to normal liver tissues [30]. However, the role of histamine in the growth of hepatoma cells is controversial. Some studies found that histamines can increase cell growth, but other studies found that histamines inhibit cell proliferation, which may depend on the different types of hepatoma cells. In this study, several H1 antihistamines exhibited antiproliferative activity in hepatoma cells. The antihistamine potency of azatadine is equal to that of cyproheptadine [31]; however, the antiproliferative activity of azatadine was less than that of cyproheptadine (Table 1). The inconsistent results suggest that the antiproliferative activity and autophagy regulation of these H1 antihistamines might not be mediated by regulating the GPCR activity of the H1 receptors. Previous studies have found that these H1 antihistamines have several other effects, including actions on muscarinic receptors, α-adrenergic receptors, serotonergic receptors, rapid delayed rectifier (IKr) channel, and other cardiac channels [28]. It is possible that the anitproliferative activity and autophagy regulation of these antihistamines might act through the above receptors or channels. However, more experiments are needed to confirm the roles of above various receptors and channels in regulating cell growth and autophagy by these H1 antihistamines. Twelve benzocycloheptene analogous drugs (Figure 1) were used and exhibited different cytotoxicity activities toward hepatoma cells (Table 1). Among these drugs, azatadine and amineptine had the weakest cytotoxicities with IC_50_ values of >50 μM, while the others exhibited better cytotoxicity. Comparing the structures and cytotoxicity of these drugs, the main structure-tricyclic ring is preferably dibenzocycloheptane rather than dibenzocycloheptene, and 7-aminoheptanoic acid is not a suitable side chain of the tricyclic ring.

Clinical studies indicated that the first-line hepatoma drug, sorafenib, combined with the antihistamine, cyproheptadine, significantly increased the mean survival time of HCC patients from 4.8 to 11 months [6]. In this study, we combined deptropine with sorfenib or erlotinib to examine whether this strategy could have synergistic effects on inhibiting hepatoma cell proliferation. Results showed that the combination index (CI) was >1.0, indicating that there were no synergistic effects when deptropine was combined with sorfenib or erlotinib in Hep3B and HepG2 cells. Each of the autophagy activators, trehalose and everolimus, as well as the autophagy inhibitors, chloroquine and wortmannin, were also combined with deptropine, but no synergistic effects were found. The activation of proteases is a necessary condition for various cell death processes. Our results indicated that deptropine might inhibit lysosomal activity (Figure 4 and Figure 5) and limited caspases activity (Figure 6). Due to the restriction of those proteases activities, detropine might not have synergistic effects when used in combination with other drugs. Autophagy can cross-talk with different types of cell death, including apoptosis, pyroptosis, necroptosis, and necrosis. Changes in the cell morphology with pyroptosis, necroptosis, and necrosis manifest as swelling, but those characteristics of apoptosis are shrinkage and extensive membrane blebbing [32,33]. After a period of deptropine treatment, cells began to detach from the cell culture dish, and then cells shrank but no bubbles occurred. The detachment was similar to anoikis, which is a kind of apoptosis induced by a lack of correct cell/extracellular matrix attachment [34]. However, deptropine-treated cells induced very limited caspase activity according to results of the Western blot and caspase activity assays. More experiments are needed to reveal the underlying molecular mechanisms of deptropine-induced cell death in hepatoma cells.

## 4. Materials and Methods

### 4.1. Materials

Amitriptyline, cyclobenzaprine hydrochloride, cyproheptadine, desloratadine, ketotifen, loratadine, nortriptyline, and pizotifen were purchased from Sigma Chemical (St. Louis, MO, USA). Azatadine was obtained from The United States Pharmacopeial Convention (USP; Rockville, MD, USA) and deptropine citrate from the European Directorate for the Quality of Medicines (EDQM; Strasbourg, France). Amineptine and rupatadine fumarate were purchased from Toronto Research Chemicals (North York, ON, Canada), and sorafenib and erlotinib were purchased from Cayman Chemical (Ann Arbor, MI, USA).

### 4.2. Cell Culture

Human hepatoma Hep3B and HepG2 cells were purchased from the Food Industry Research and Development Institute (Hsinchu, Taiwan), and cultured in high-glucose Dulbecco’s modified Eagle medium (DMEM) (Thermo Fisher Scientific Inc., Waltham, MA, USA) supplemented with 10% heat-inactivated fetal bovine serum (FBS), 1% non-essential amino acids, 1% sodium pyruvate, and 1% L-glutamine, and maintained in a humidified incubator at 37 °C with 5% CO_2_.

### 4.3. 3-(4,5-Dimethylthiazol-2-yl)-2,5-Diphenyltetrazolium Bromide (MTT) Assay

Cells (8 × 10^3^) were cultured in a 96-well plate for 18–24 h and then treated with drugs for another 24–72 h. After the cell viability was determined, the medium was removed from each well, and another 200 μL of fresh medium, as well as 50 μL of MTT (2 mg/mL), was added at 37 °C in the dark. After 4 h, the medium was removed, and 100 μL of dimethyl sulfoxide (DMSO) and 12.5 μL of Sorensen’s glycine buffer (0.1 M glycine, 0.1 M NaCl, pH 10.5) were added, and then the absorbance was measured at optical density (OD) 570 nm by an enzyme-linked immunosorbent assay (ELISA) plate reader [35].

### 4.4. Western Blot Analysis

Equal amounts of total cellular protein (10–30 μg) were resolved by 10% sodium dodecylsulfate (SDS)-polyacrylamide gel electrophoresis (PAGE) and transferred onto a polyvinylidene difluoride (PVDF) membrane (Millipore, Bedford, MA, USA) as described previously [36]. The membrane was then incubated with the following primary antibodies: anti-PARP (#9532), anti-caspase-3 (#9662), anti-caspase-9 (#9508), anti-caspase-8 (#4790), anti-light chain 3B (LC3B) (#2775), anti-phospho (T308)-Akt (#4056) (Cell Signaling Technology, Danvers, MA, USA), anti-PKR-like endoplasmic reticular kinase (PERK) (sc-377400), anti-phospho (Thr981)-PERK (sc-32577), anti-eIF2α (sc-11386), anti-phospho-eIF2α (sc-101670), anti-phospho-adenosine 5′-monophosphate-activated protein kinase α (AMPKα) (sc-398861), anti-Akt (sc-377457), anti-cathepsin L (sc-390385) (Santa Cruz Biotechnology, Santa Cruz, CA, USA), anti-LC3B (GTX100240), anti-SQSTM1/p62 (GTX629890), anti-autophagy-related 7 (ATG7) (GTX 113613), anti-vacuolar protein sorting 34 (VPS34) (GTX129528), anti-GAPDH (GTX100118), and anti-Grp78 (GTX113340) (GeneTex, Alton Pkwy Irvine, CA, USA). The blots were incubated with the primary antibodies anti-phospho-eIF2α and anti-phospho (Thr981)-PERK at a dilution of 1:500, anti-Grp78 at a dilution of 1:10,000, and the others at a dilution of 1:1000. Membranes were subsequently incubated with an anti-mouse or anti-rabbit immunoglobulin G (IgG) secondary antibody conjugated to horseradish peroxidase (Santa Cruz Biotechnology) at a dilution of 1:5000 and visualized using enhanced chemiluminescence kits (Amersham, Arlington, IL, USA).

### 4.5. Transient Transfection

Cells (6 × 10^4^) were seeded onto glass coverslips in 12-well plates and transfected with the FUW mCherry-GFP-LC3B plasmid [37] by LipofectamineTM 3000 reagent (Life Technologies, Taiwan Brand, Taipei, Taiwan). Green fluorescent protein (GFP) and mCherry fluorescence were observed and photographed with the Confocal Spectral Microscope Imaging System (Leica TCS SP5, Wetzlar, Germany). FUW mCherry-GFP-LC3 was a gift from Anne Brunet (Addgene plasmid #110060; http://n2t.net/addgene:110060; RRID: Addgene_110060).

### 4.6. Immunofluorescence Staining

Cells were seeded onto glass coverslips in 12-well plates, and immunofluorescence staining was performed according to the manufacturer’s instructions [38]. Briefly, cells were fixed with 4% paraformaldehyde for 10 min and permeabilized in 0.5% Triton X-100 (Sigma Chemical) for 15 min. After a phosphate-buffered saline (PBS) wash, cells were incubated with blocking solution (5% bovine serum albumin) at room temperature for 1 h, a primary anti-LC3B antibody (#2775 from Cell Signaling Technology; 1:200 dilution) at 4 °C for 18 h, and then secondary anti-rabbit IgG (CF^®^488-conjugated; Biotium Corporation, Fremont, CA, USA) at room temperature for 1 h under continuous stirring (25 rpm). Cells were then fixed in Prolong Gold Antifade Mountant (Thermo Fisher Scientific Taiwan, Taipei, Taiwan) with 4′,6-diamidino-2-phenylindole (DAPI) (Biotium Inc., Fremont, CA, USA) to visualize cell nuclei and photographed using the TCS SP5 Confocal Spectral Microscope Imaging System.

### 4.7. Caspase Activity Assay

Caspase -3, -8, and -9 activities were determined according to the manufacturer’s instructions (BioVision, Milpitas, CA, USA). Briefly, cells were washed with cold PBS twice, and lysed in cell lysis buffer. The supernatant (cytosolic extract, 100 μg) was incubated with 200 μM of substrate, Asp-Glu-Val-Asp-*p*-nitroaniline (DEVD-pNA for caspase-3), Ile-Glu-Thr-Asp-*p*-nitroaniline (IETD-pNA for caspase-8), or Leu-Glu-His-Asp-*p*-nitroaniline (LEHD-pNA for caspase-9) in reaction buffer (50 mM HEPES at pH 7.4, 100 mM NaCl, 0.1% CHAPS, 10 mM dithiothreitol (DTT), 1 mM ethylenediaminetetraacetic acid (EDTA), and 10% glycerol) at 37 °C for 1 h in the dark. The cleaved chromophore pNA was measured using a microtiter plate reader at 405 nm [39].

### 4.8. Antitumor Nude Mice Experiment

Six-week-old male Balb/cAnN-Foxn1 nude mice (BioLasco Taiwan, Taipei, Taiwan) were kept in an animal facility for 1–2 weeks before use. All mice were subcutaneously inoculated with 10^7^ of Hep3B cells and an equal volume of Matrigel™ Basement Membrane Matrix (BD Biosciences Taiwan, Taipei, Taiwan). At 19 days after transplantation, tumor-bearing mice (seven or eight mice/group) were intraperitoneally (i.p.) treated with 20 μL DMSO (vehicle) or deptropine (2.5 mg/kg) three times a week for 3 weeks. The tumor volume was estimated according to the following formula: tumor volume (mm^3^) = L × W2/2, where L is the length and W is the width, and the tumor weight was determined after mice were sacrificed [40]. The animal study was approved by the Institutional Animal Care and Use Committee of Taipei Medical University (LAC-2017-0047).

### 4.9. Statistical Analysis

Data are presented as the mean ± standard error (SE) for the indicated number of independently performed experiments. Statistical analyses were performed using one-way Student’s *t*-test, and differences were considered significant at *p* < 0.05.

## 5. Conclusions

Our findings of inhibition of autophagy and induction of hepatoma cell death by deptropine, suggesting that deptropine and other structural-analogue drugs might be potential drugs for clinical treatment of hepatoma.

## Figures and Tables

**Table 1:** Cytotoxic effects of 12 benzocycloheptene analogous drugs toward human hepatoma cells.

Drug.	Hep3B IC_50_ (μM)	HepG2 IC_50_ (μM)
Azatadine	>50.00	>50.00
Cyproheptadine	23.00 ± 0.28	29.39 ± 0.58
Deptropine	9.98 ± 0.12	9.75 ± 0.11
Ketotifen	18.46 ± 0.11	23.85 ± 0.18
Pizotifen	31.73 ± 0.21	31.69 ± 0.16
Rupatadine	13.35 ± 0.14	14.68 ± 0.12
Desloratadine	21.59 ± 0.20	32.09 ± 0.64
Loratadine	18.27 ± 0.22	21.46 ± 0.19
Cyclobenzaprine	23.98 ± 0.33	26.79 ± 0.45
Amineptine	>50.00	>50.00
Amitriptyline	21.00 ± 0.20	15.15 ± 0.21
Nortriptyline	13.22 ± 0.27	17.10 ± 0.21

Hep3B and HepG2 cells were treated with different concentrations of the 12 benzocycloheptene analogous drugs for 72 h, and the cell viability was determined by an MTT assay. Each 50% inhibitory concentration (IC_50_) is presented as the mean ± standard error of three independent experiments.

## Supplementary Materials

The following are available online at https://www.mdpi.com/2072-6694/12/6/1610/s1, Figure S1: Uncropped Western Blots of Figure 3a., Figure S2: Uncropped Western Blots of Figure 3b, Figure S3: Uncropped Western Blots of Figure 3c, Figure S4: Uncropped Western Blots of Figure 5, Figure S5: Uncropped Western Blots of Figure 6a.
