# Supplementary material for: The Antihistamine Deptropine Induces Hepatoma Cell Death through Blocking Autophagosome-Lysosome Fusion
Source: Cancers (Basel). 2020 Jun 18;12(6):1610. doi: 10.3390/cancers12061610 (PMC7352610; doi:10.3390/cancers12061610)
Supplement: Supplementary file 1 [file cancers-12-01610-s001.pdf]

cancers

Article

The Antihistamine Deptropine Induces Hepatoma Cell Death Through Blocking Autophagosome-Lysosome Fusion

Yu-Chih Liang, Chi-Ching Chang, Ming-Thau Sheu, Shyr-Yi Lin, Chia-Chen Chung, Chang-Ting Teng and Fat-Moon Suk

Supplementary Material

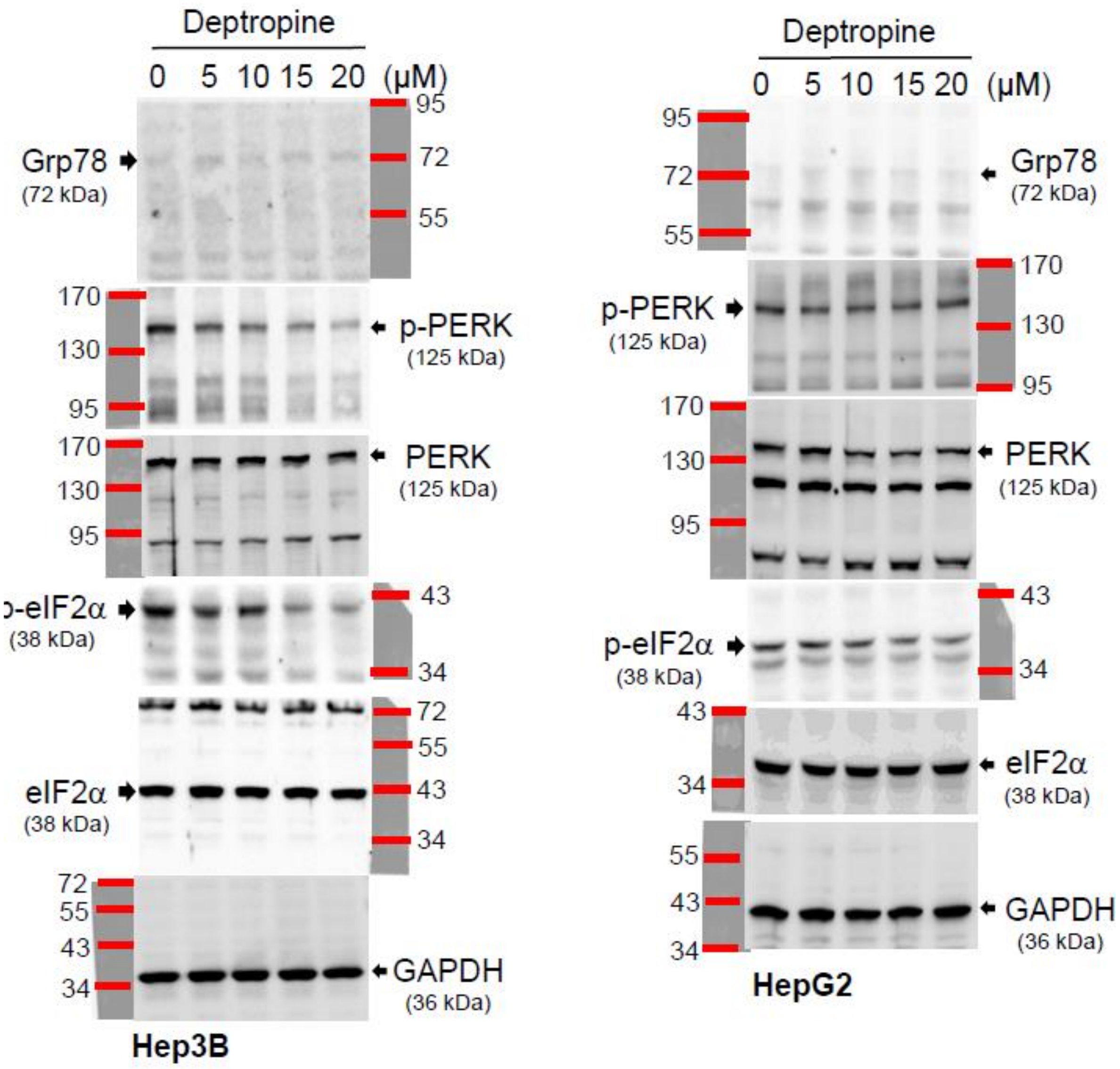

Figure S1. Uncropped Western Blots of Figure 3a.

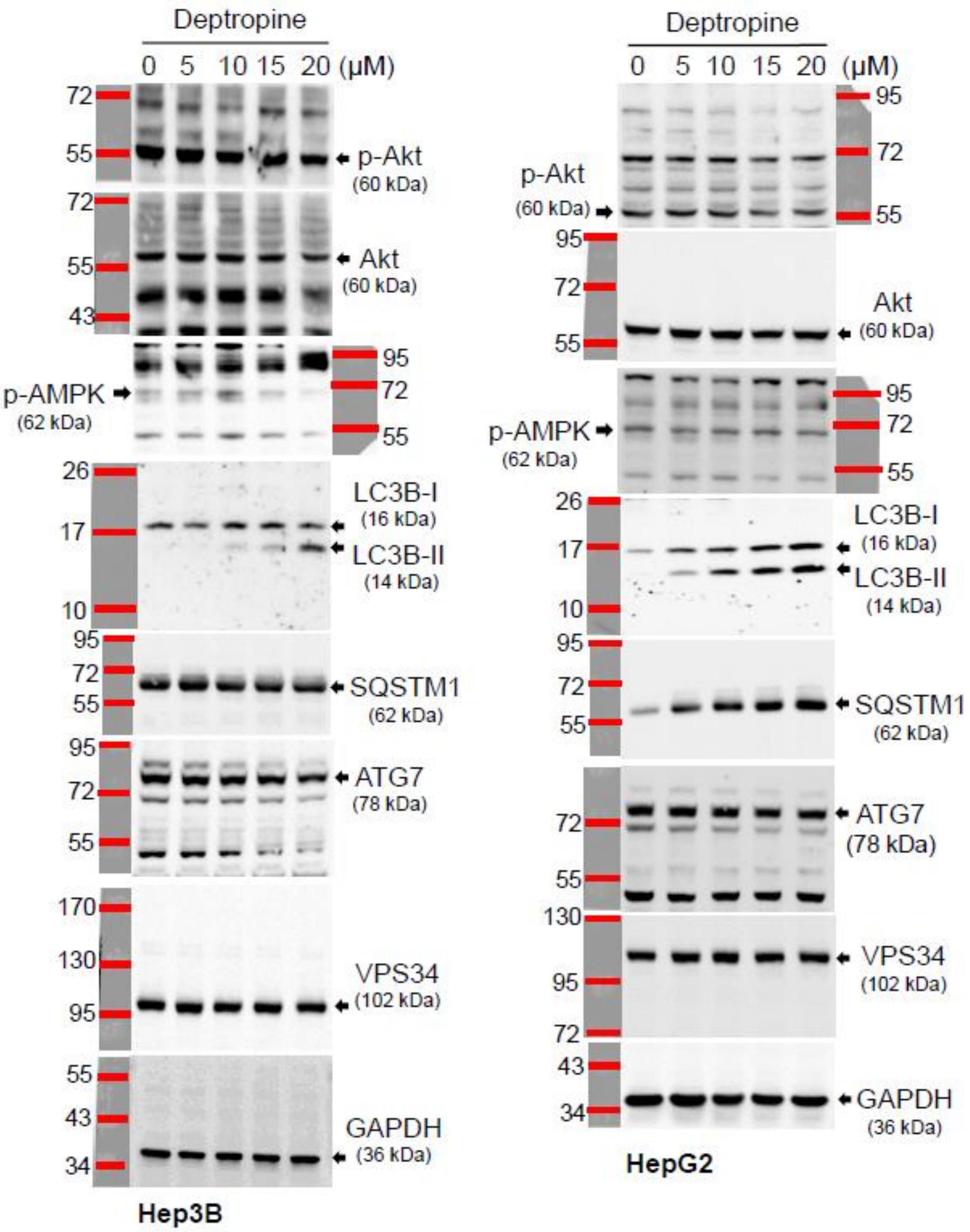

Figure S2. Uncropped Western Blots of Figure 3b.

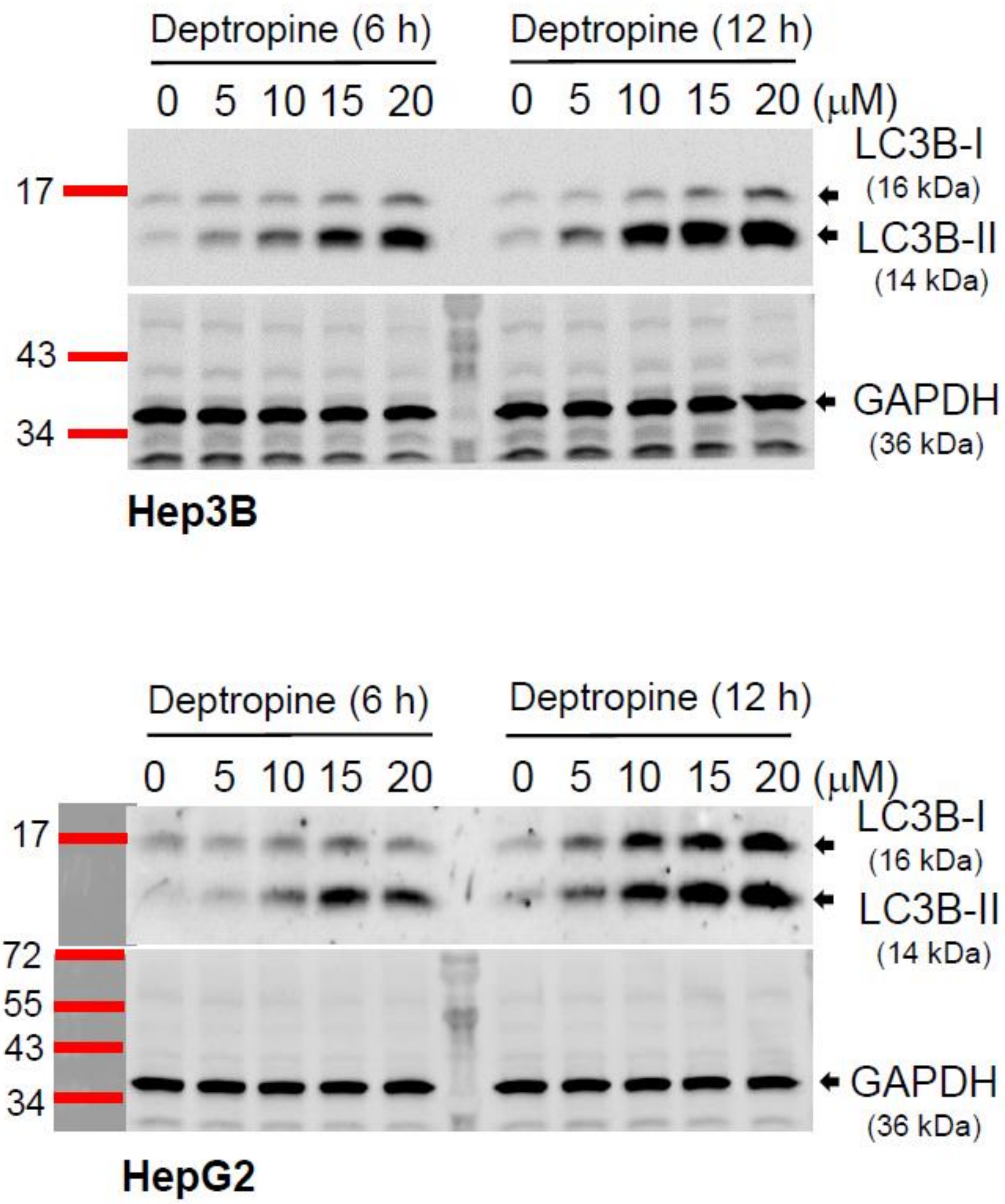

Figure S3. Uncropped Western Blots of Figure 3c.

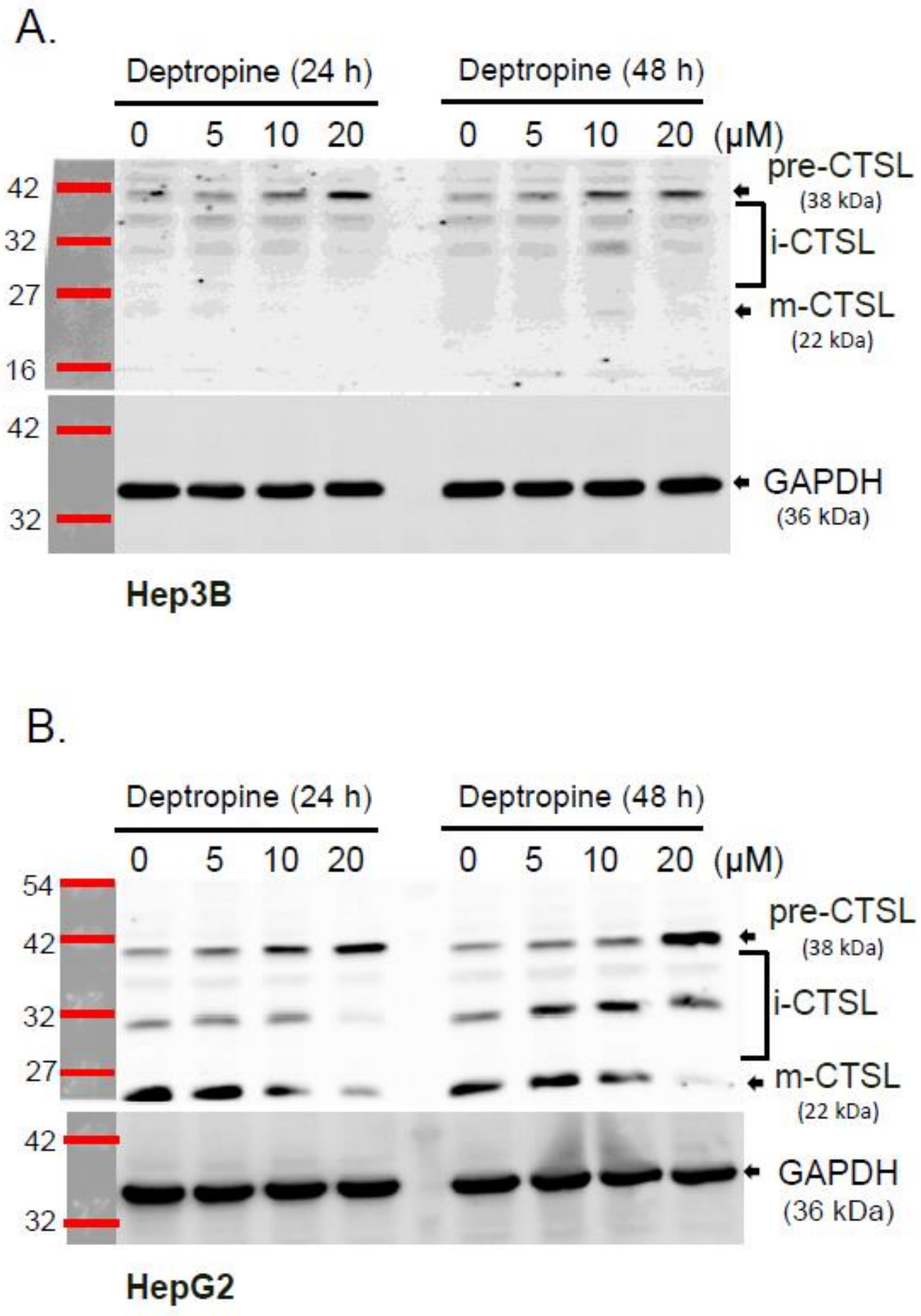

Figure S4. Uncropped Western Blots of Figure 5. (**A**) Hep3B cells and (**B**) HepG2 cells.

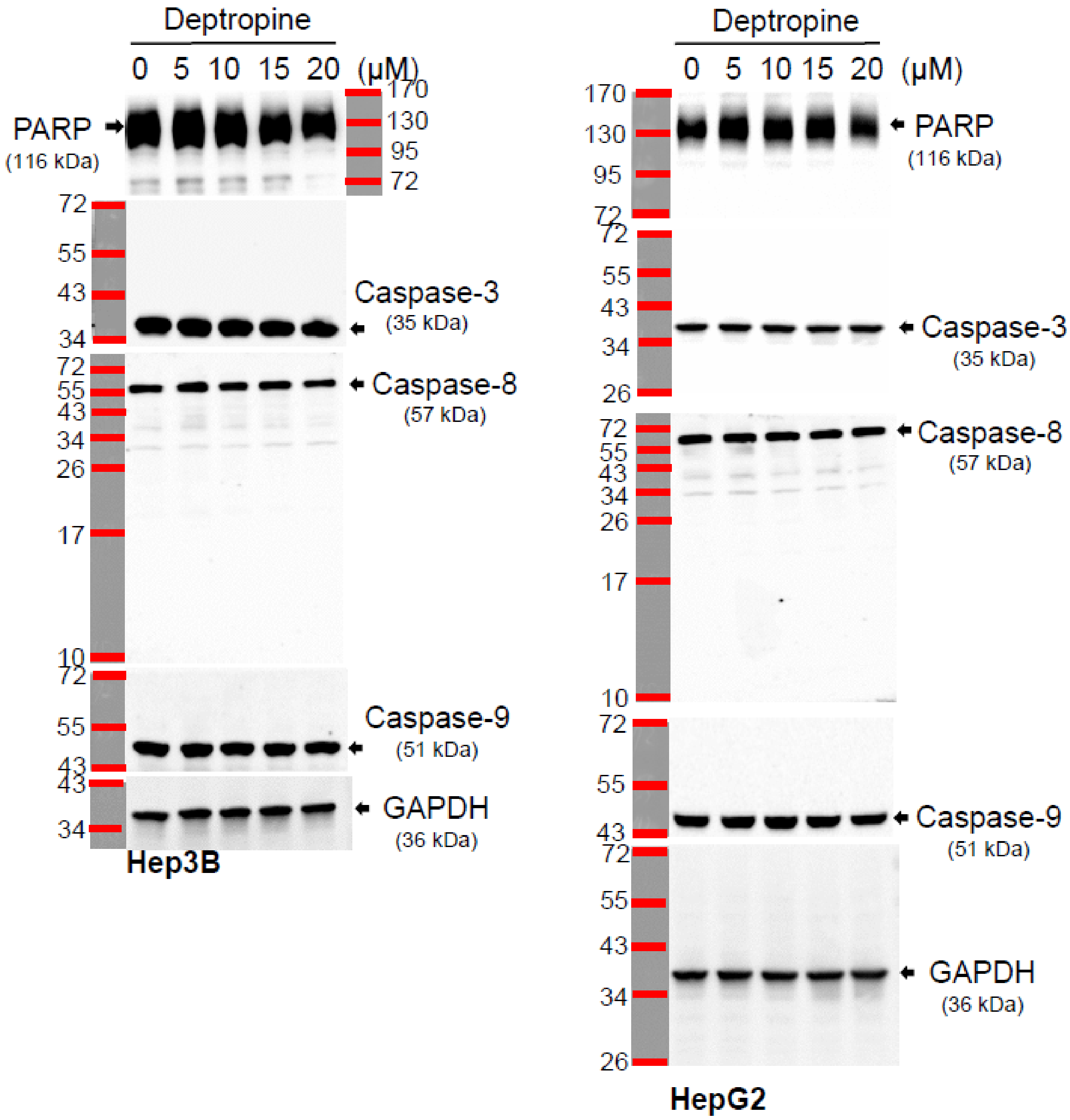

Figure S5. Uncropped Western Blots of Figure 6a.
